# Supplementary material for: Genomic epidemiology reveals geographical clustering of multidrug-resistant Escherichia coli ST131 associated with bacteraemia in Wales
Source: Nat Commun. 2024 Feb 14;15:1371. doi: 10.1038/s41467-024-45608-1 (PMC10866875; doi:10.1038/s41467-024-45608-1)
Supplement: Supplementary file 1 — Supplementary Information [file 41467_2024_45608_MOESM1_ESM.pdf]

**SUPPLEMENTARY APPENDIX**

# Genomic epidemiology reveals geographical clustering of multidrug-resistant *Escherichia coli* ST131 associated with bacteraemia in Wales

Rhys T. White[1,2,3], Matthew J. Bull[4,5], Clare R. Barker[4], Julie M. Arnott[6], Mandy Wootton[5], Lim S. Jones[5], Robin A. Howe[5], Mari Morgan[6], Melinda M. Ashcroft[7], Brian M. Forde[2,8], Thomas R. Connor[4,9*], Scott A. Beatson[1,2,3*]

[1]School of Chemistry and Molecular Biosciences, The University of Queensland, Brisbane, Queensland 4072, Australia

[2]Australian Infectious Disease Research Centre, The University of Queensland, Brisbane, Queensland 4072, Australia

[3]Australian Centre for Ecogenomics, The University of Queensland, Brisbane, Queensland 4072, Australia

[4]Microbiomes, Microbes and Informatics Group, Organisms and Environment Division, School of Biosciences, Cardiff University, Cardiff, Wales CF10 3AX, United Kingdom

[5]Public Health Wales Microbiology, University Hospital Wales, Cardiff, Wales CF14 4XW, United Kingdom

[6]Healthcare Associated Infection, Antimicrobial Resistance & Prescribing Programme (HARP), Public Health Wales, 2 Capital Quarter, Tyndall Street, Cardiff, Wales CF10 4BZ, United Kingdom

[7]Department of Microbiology and Immunology, The University of Melbourne at The Peter Doherty Institute for Infection and Immunity, Melbourne, Victoria, Australia

[8]The University of Queensland, UQ Centre for Clinical Research (UQCCR), Royal Brisbane & Women's Hospital Campus, Herston, Queensland 4029, Australia

[9]Public Health Genomics Programme, Public Health Wales, 2 Capital Quarter, Tyndall Street, Cardiff, Wales CF10 4BZ, United Kingdom

Correspondence and requests for materials should be addressed to T.R.C. (email: connortr@cardiff.ac.uk) or to S.A.B. (email: s.beatson@uq.edu.au)

## <u>This file includes:</u>

Supplementary Methods

Supplementary Figure S1. Deaths registered in each calendar year in Wales involving *Escherichia coli* septicaemia between 2001 and 2015.

Supplementary Figure S2. Population estimates by lower layer super output areas, 2014.

Supplementary Figure S3. Nucleotide comparisons between key sequence type determining housekeeping genes within the reference chromosome EC958.

Supplementary Figure S4. Maximum likelihood phylogenetic analysis representing global *Escherichia coli* sequence type (ST)131.

Supplementary Figure S5. Maximum parsimony phylogeny of clade C/*H*30 *Escherichia coli* sequence type (ST)131 isolates plotted against *β*-lactam resistance complement.

Supplementary Figure S6. Evolutionary reconstruction of clade C/*H*30 *Escherichia coli* sequence type (ST)131.

## SUPPLEMENTARY METHODS

### Quality control of sequence data for the 157 Welsh strains

The FastQC package v0.11.8 (http://www.bioinformatics.babraham.ac.uk/projects/fastqc/, accessed 22 January 2024) was used to generate quality statistics for the paired-end reads, which were aggregated into a single report and visualised using MultiQC v1.7[1]. Kraken v2.0.7-beta[2] was then used to screen the raw Illumina sequencing data for contamination against the National Center for Biotechnology Information (NCBI) RefSeq database[3]. Raw reads were filtered using Trimmomatic v0.36[4] by removing low-quality bases and read pairs together with Illumina adaptor sequences (settings: LEADING:10 TRAILING:10 MINLEN:50 HEADCROP:10). The average sequence coverage depth was estimated using the Burrows–Wheeler Aligner v0.7.15[5]; SAMtools v1.2[6]; Picard v2.7.1 (https://github.com/broadinstitute/picard, accessed 22 January 2024); the Genome Analysis Tool Kit v3.2-2 (GATK)[7,8]; BEDTools v2.18.2[9]; and SNPEff v4.1[10] as implemented in SPANDx v3.2[11]. In brief, the trimmed reads were mapped to the complete chromosome of *E. coli* ST131 strain EC958 (GenBank: HG941718); which was isolated from the urine of an 8-year-old girl presenting in the community in March 2005 in the United Kingdom[12]. We identified and excluded the sequence data for 15 isolates from further analysis based on the sequencing coverage below 20-fold (Supplementary Data 3).

### *In silico* gene typing

MLST v2.19.0 (https://github.com/tseemann/mlst, accessed 22 January 2024) with default settings was used to characterise the Multi-Locus Sequence Type (MLST) of the 142 strains by querying the high-quality draft assemblies against the *E. coli* MLST allelic profiles hosted on PubMLST[13,14]. ABRicate v0.9.7 (https://github.com/tseemann/abricate, accessed 22 January 2024) was used to screen the high-quality draft assemblies for O and H-antigens, acquired antimicrobial resistance genes, and bacterial plasmid replicons using the EcOH[15], ARG-ANNOT[16], and PlasmidFinder[17] databases, respectively (last updated 07 September 2019). PointFinder[18] was used to screen high-quality draft assemblies for chromosomal point mutations, particularly in the QRDR of *gyrA*, *gyrB*, *parC*, and *parE* genes[19,20]. The K-antigen and *fimH* allele were characterised using Kaptive v0.4[21] (default settings) against a custom *E. coli* database comprising of known capsule antigens from complete genomes available on NCBI and FimTyper 1.0 (https://cge.food.dtu.dk/services/FimTyper/, accessed 22 January 2024) with default parameters, respectively.

**Assembly-based ST131 phylogeny**

We initially aimed to place our Welsh isolates (*n*=142) within the context of ST131 isolates sampled globally (*n*=208). The generation of a core-genome (based on homology) multi-alignment, and identification of single-nucleotide polymorphisms (SNPs) was performed with Parsnp v1.2[22], utilising the PhiPack recombination filter[23]. A total of 13,854 (13,758 non-recombinant) core-genome SNPs relative to the reference chromosome EC958 were identified from a 2,575,140 bp core-genome alignment. Finally, RAxML v8.2.10[24] with GTR-GAMMA correction generated a maximum likelihood (ML) phylogeny thorough optimisation of the 20 distinct randomised maximum parsimony trees generated from the 13,758 non-recombinant core-genome SNP alignment.

**Identifying and removing strain mixtures from the clade C ST131 phylogeny**

A total of 245 strains representing our previously published collection (*n*=127, including 16 complete genomes (including EC958)) and our Welsh collection (*n*=102) were identified as either sub-clade C1 or C2 ST131 as described above. To assess the 245 strains for the presence of strain mixtures, paired-end reads were mapped onto the chromosome of EC958 using SPANDx to generate annotated SNPs and insertions and deletions (INDELs) matrices. Heterozygous SNPs in each genome were identified from GATK UnifiedGenotyper VCF output. A total of seven genomes were classified as strain mixtures based on the orthologous SNP alignment containing ~3% or more heterozygous SNPs sites and were subsequently removed from the dataset, leaving 238 genomes. These includes strains: JJ1908 (*n*=309/7,592, 4.1%); P146EC (*n*=299/7,592, 3.9%); S43EC (*n*=289/7,592, 3.8%); ZH164 (*n*=285/7,592, 3.8%); S39EC (*n*=278/7,592, 3.7%); G150 (*n*=272/7,592, 3.6%); and S30EC (*n*=210/7,592, 2.8%).

## SUPPLEMENTARY FIGURES

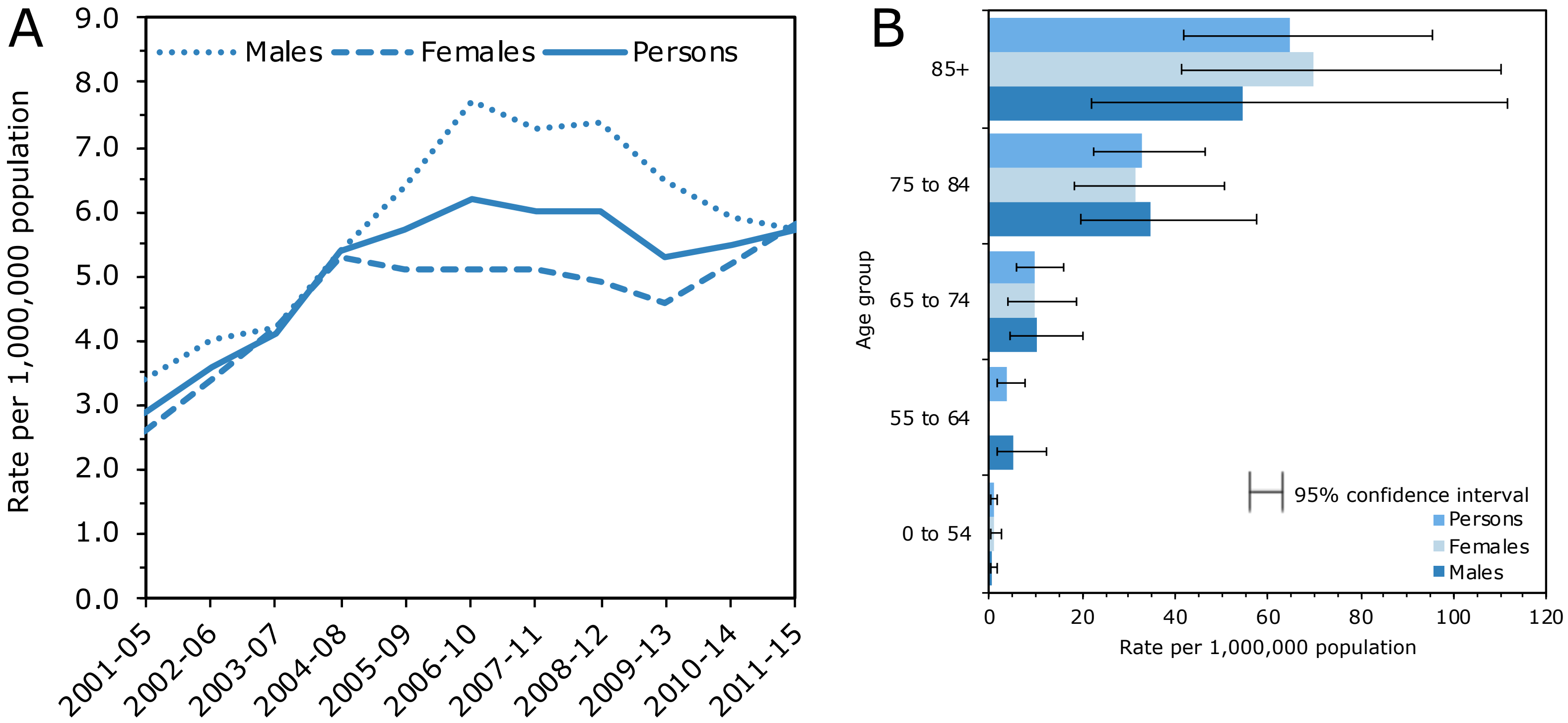


**Supplementary Figure S1. Deaths registered in each calendar year in Wales involving *Escherichia coli* septicaemia between 2001 and 2015.** Due to small numbers of deaths for individual years, deaths were pooled into 5-year periods to calculate more robust rates. Figures are based on postcode boundaries as of May 2016 and exclude deaths of non-residents. Statistically significant differences between rates were assessed using 95% confidence intervals (CI). (A) Age-standardised mortality 5-year rolling rates per million population. 95% CI are not displayed as there is no significant difference between sexes. (B) Age-specific mortality 5-year rolling rates per million population, 2011 to 2015. Adapted from "Deaths involving *E. coli* septicaemia, deaths registered in Wales between 2001 and 2015 [Online]" by the Office for National Statistics. Available at: https://www.ons.gov.uk/peoplepopulationandcommunity/birthsdeathsandmarriages/deaths/adhocs/006005deathsinvolvingecolisepticaemiadeathsregisteredinwalesbetween2001and2015 [Accessed 31st January 2017]. (2016). Copyright © 2016 by Office for National Statistics.

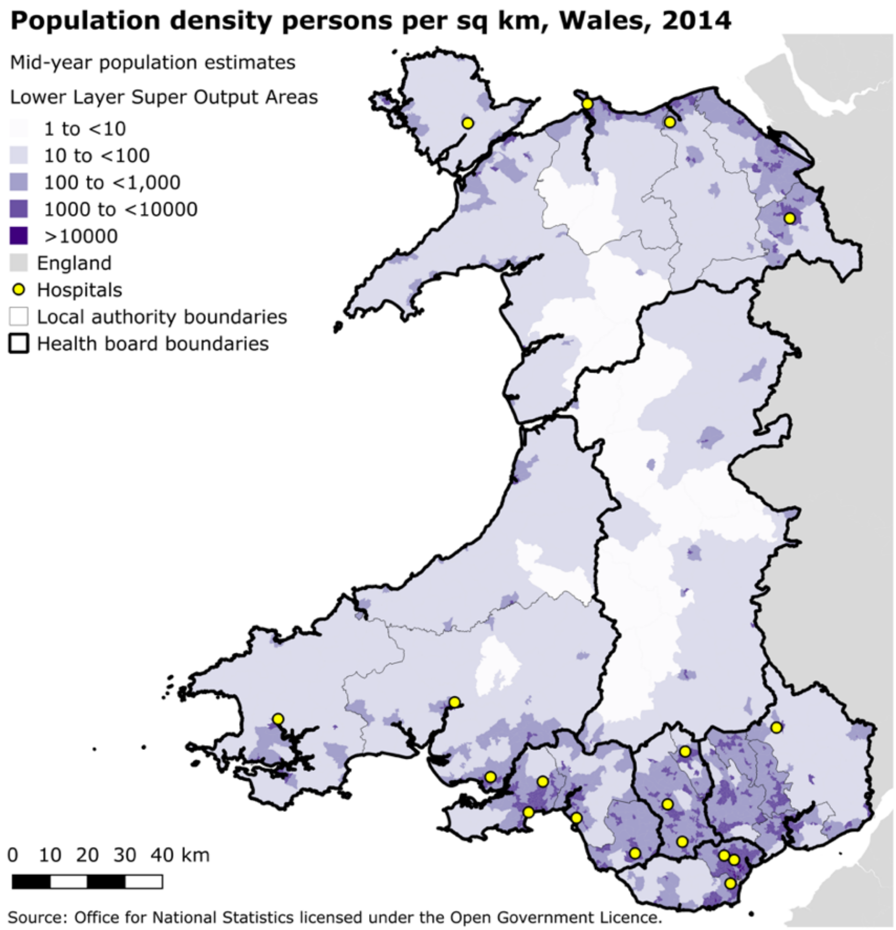


**Supplementary Figure S2. Population estimates by lower layer super output areas, 2014.** The population of Wales is expressed as population per square kilometre of land. The map was produced using QGIS v3.24.3-Tisler (http://www.qgis.org, accessed 22 January 2024), using the layers Local (Unitary) Authority boundaries for Wales, Local Health Boards Boundaries in Wales, and Lower layer Super Output Areas (LSOA) in Wales (https://www.data.gov.uk/, accessed 22 January 2024). Mid-year population estimates were retrieved from "Table SAPE20DT11: Mid-2014 Population Density for Lower Layer Super Output Areas in England and Wales - National Statistics [Online]" by the Office for National Statistics. Available at: https://www.ons.gov.uk/peoplepopulationandcommunity/populationandmigration/populationestimates/datasets/lowersuperoutputareapopulationdensity [Accessed 28 April 2019]. (2018). Copyright © 2018 by Office for National Statistics.

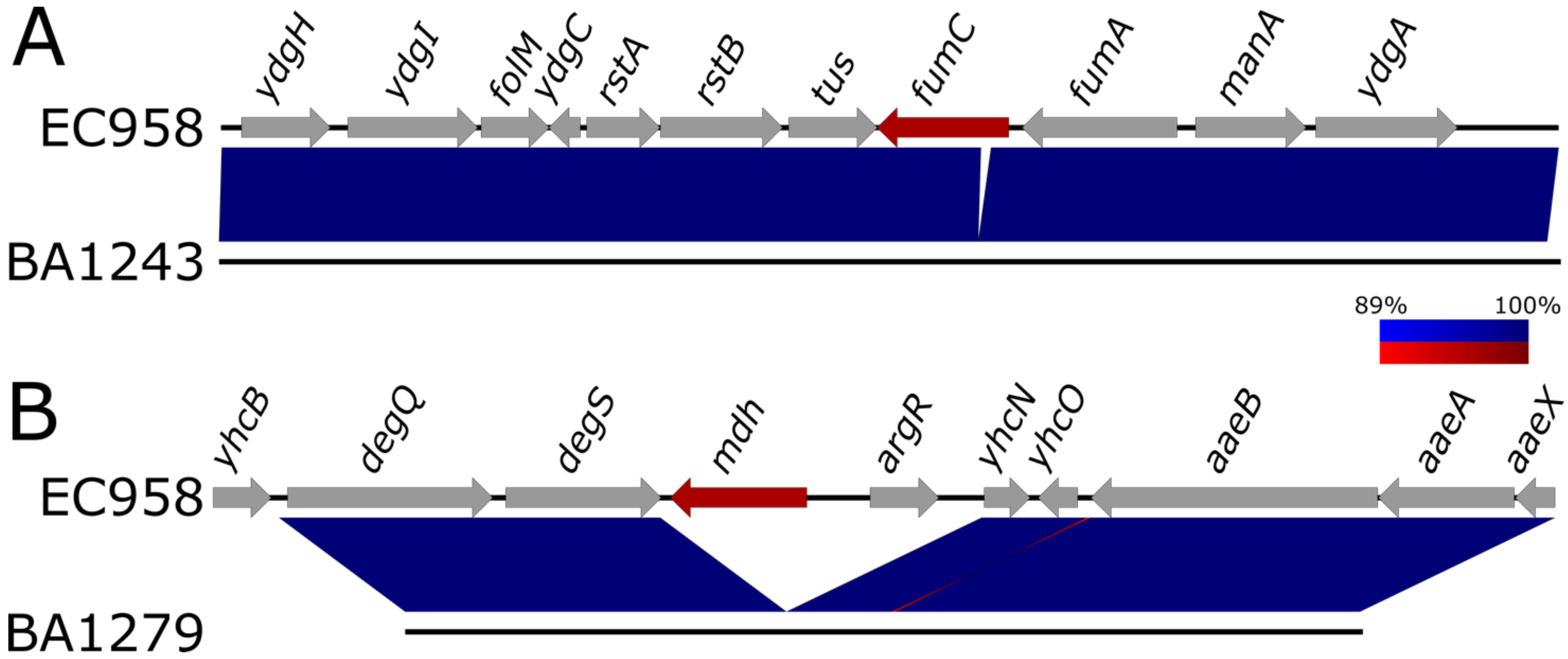


**Supplementary Figure S3. Nucleotide comparisons between key sequence type determining housekeeping genes within the reference chromosome EC958**. Blue shading indicates nucleotide identity (red, inverted regions) between sequences according to BLASTn (89 to 100%). Key housekeeping genes are indicated in red, other CDSs in grey. Image created using EasyFig[25].

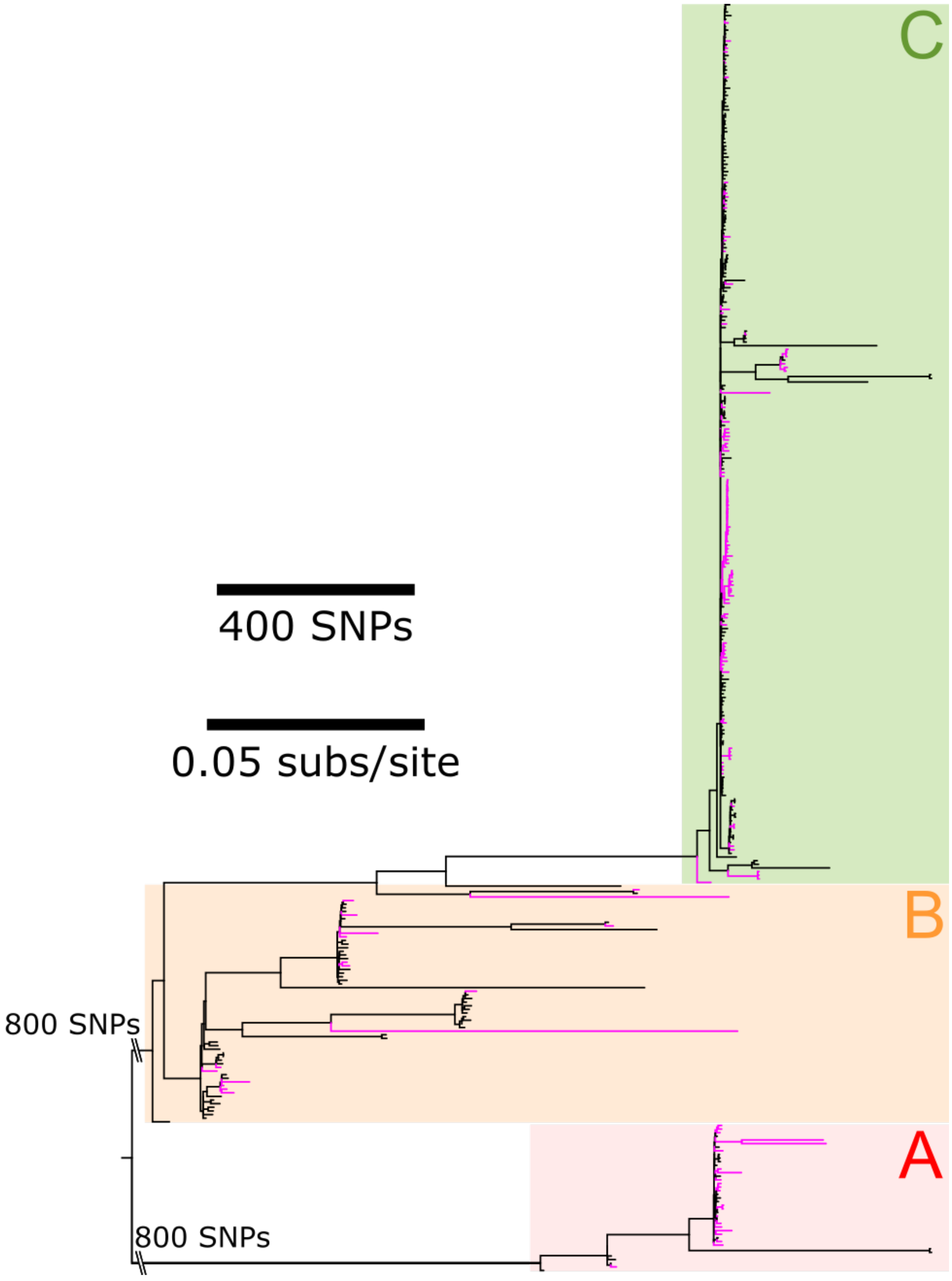


**Supplementary Figure S4. Maximum likelihood phylogenetic analysis representing global *Escherichia coli* sequence type (ST)131.** Phylogeny is inferred from 13,758 non-recombinant core-genome single nucleotide polymorphisms (SNPs) relative to the reference chromosome EC958. SNPs were identified with Parsnp with the PhiPack recombination filter and represent a 2,575,140 bp core-genome. The phylogenetic tree is rooted according to the midpoint. Branch lengths represent SNP distances or nucleotide substitutions per site as indicated by the scale bars. The major ST131 phylogenetic clades are indicated: A = red, B = yellow, and C = green. Strains from Wales (*n*=142) are highlighted in pink.

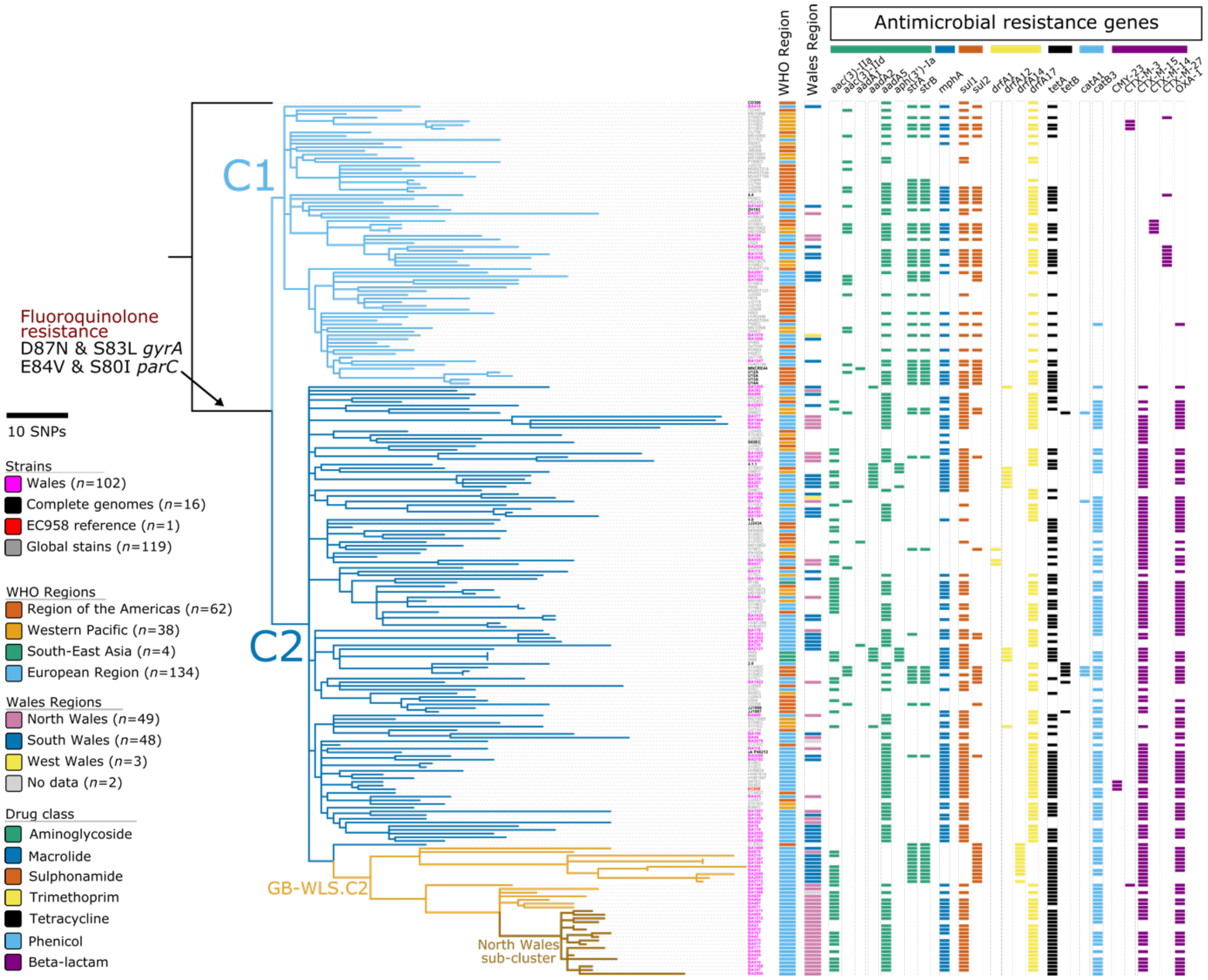


**Supplementary Figure S5. Maximum parsimony phylogeny of clade C *Escherichia coli* sequence type (ST)131 isolates plotted against antimicrobial resistance complement.** Phylogeny inferred from 4,142 non-recombinant orthologous biallelic core-genome single-nucleotide polymorphisms (SNPs) from 238 strains. Moderate recombination SNP density filtering in SPANDx (excluded regions with ≥3 SNPs in a 100 bp window). SNPs are derived from read mapping to the reference chromosome EC958 (GenBank HG941718). Phylogenetic trees are rooted according to the CD306 (GenBank: CP013831) outgroup. Branch lengths represent SNP distances indicated by the scale bar. The consistency index is 0.99.

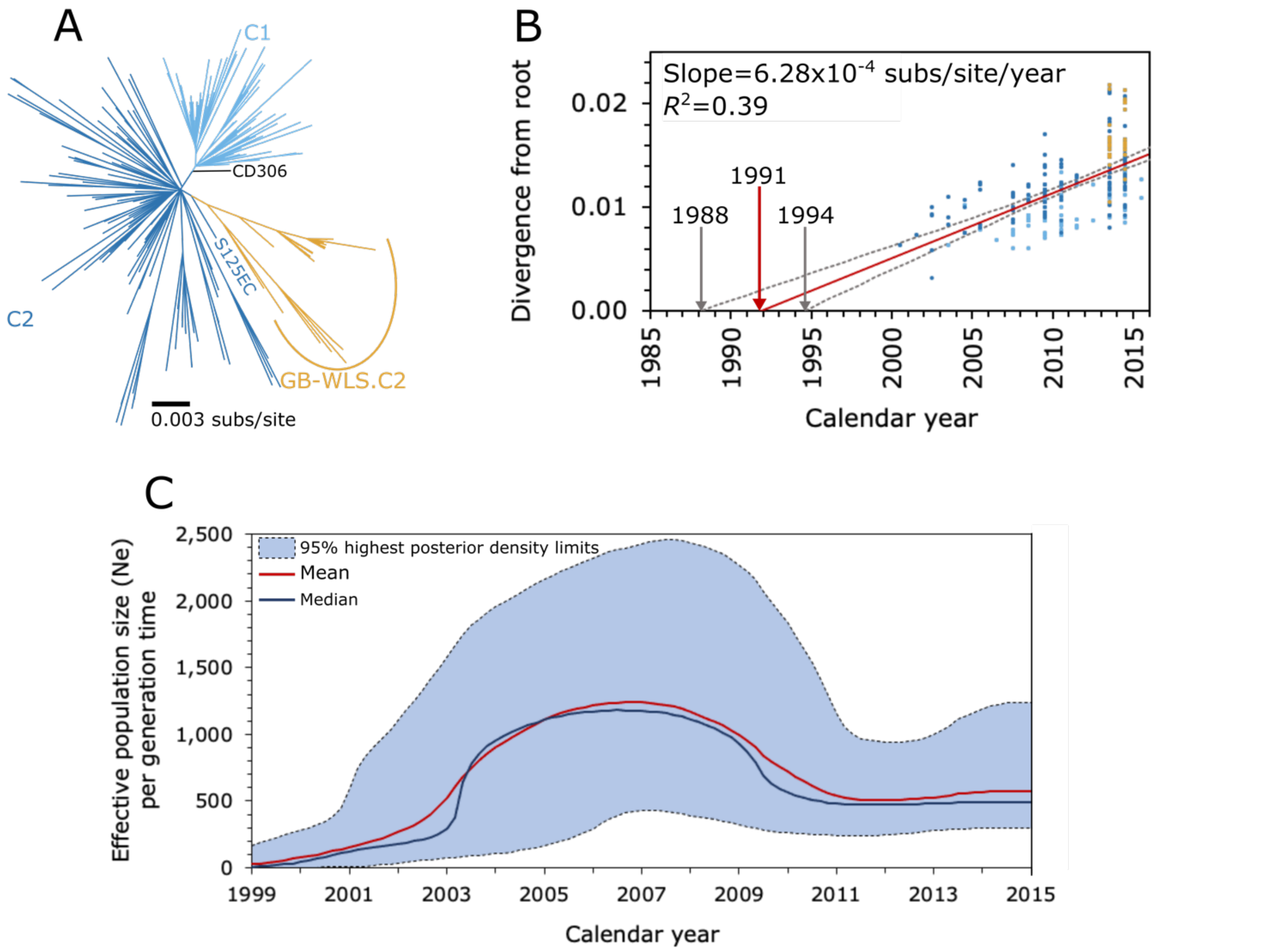


**Supplementary Figure S6. Evolutionary reconstruction of clade C *Escherichia coli* sequence type (ST)131.** (A) Maximum likelihood phylogeny of 238 clade C isolates inferred from 4,142 non-recombinant orthologous biallelic core-genome single-nucleotide polymorphisms (SNPs). Moderate recombination SNP density filtering in SPANDx (excluded regions with ≥3 SNPs in a 100 bp window). SNPs are derived from read mapping to the reference chromosome EC958 (GenBank: HG941718). Branch lengths represent nucleotide substitutions per site as indicated by the scale bar. (B) Linear regression of root-to-tip genetic distance plotted against year of collection as implemented in TempEST. The substitution rate for the tree in A is indicated by the slope of the solid red regression line supported by 95% confidence intervals (grey dashed lines). Trees in A and B were rooted according to the *E. coli* CD306 (GenBank: CP013831) outgroup. (C) A Bayesian Skyline plot showing the predicted demographic changes of the ST131 clade C population since 1999.

# REFERENCES FOR SUPPLEMENTARY APPENDIX


1. **Ewels P, Magnusson M, Lundin S, Kaller M**. MultiQC: summarize analysis results for multiple tools and samples in a single report. *Bioinformatics* 2016;32:3047-3048 doi: 10.1093/bioinformatics/btw354

2. **Wood DE, Salzberg SL**. Kraken: ultrafast metagenomic sequence classification using exact alignments. *Genome Biology* 2014;15:1-12 doi: 10.1186/gb-2014-15-3-r46

3. **Sayers EW, Barrett T, Benson DA, Bryant SH, Canese K, Chetvernin V, *et al***. Database resources of the National Center for Biotechnology Information. *Nucleic Acids Research* 2010;39:D38–D51 doi: 10.1093/nar/gkq1172

4. **Bolger AM, Lohse M, Usadel B**. Trimmomatic: a flexible trimmer for Illumina sequence data. *Bioinformatics* 2014;30:2114-2120 doi: 10.1093/bioinformatics/btu170

5. **Li H, Durbin R**. Fast and accurate short read alignment with Burrows-Wheeler transform. *Bioinformatics* 2009;25:1754-1760 doi: 10.1093/bioinformatics/btp324

6. **Li H, Handsaker B, Wysoker A, Fennell T, Ruan J, Homer N, *et al***. The Sequence Alignment/Map format and SAMtools. *Bioinformatics* 2009;25:2078-2079 doi: 10.1093/bioinformatics/btp352

7. **McKenna A, Hanna M, Banks E, Sivachenko A, Cibulskis K, Kernytsky A, *et al***. The Genome Analysis Toolkit: a MapReduce framework for analyzing next-generation DNA sequencing data. *Genome Research* 2010;20:1297-1303 doi: 10.1101/gr.107524.110

8. **DePristo MA, Banks E, Poplin R, Garimella KV, Maguire JR, Hartl C, *et al***. A framework for variation discovery and genotyping using next-generation DNA sequencing data. *Nature Genetics* 2011;43:491-498 doi: 10.1038/ng.806

9. **Quinlan AR, Hall IM**. BEDTools: a flexible suite of utilities for comparing genomic features. *Bioinformatics* 2010;26:841-842 doi: 10.1093/bioinformatics/btq033

10. **Cingolani P, Platts A, Wang le L, Coon M, Nguyen T, Wang L, *et al***. A program for annotating and predicting the effects of single nucleotide polymorphisms, SnpEff: SNPs in the genome of *Drosophila melanogaster* strain $w^{1118}$; iso-2; iso-3. *Fly* 2012;6:80-92 doi: 10.4161/fly.19695

11. **Sarovich DS, Price EP**. SPANDx: a genomics pipeline for comparative analysis of large haploid whole genome re-sequencing datasets. *BMC Research Notes* 2014;7:618 doi: 10.1186/1756-0500-7-618

12. **Totsika M, Beatson SA, Sarkar S, Phan MD, Petty NK, Bachmann N, *et al***. Insights into a multidrug resistant *Escherichia coli* pathogen of the globally disseminated ST131 lineage: genome analysis and virulence mechanisms. *PLOS ONE* 2011;6 doi: 10.1371/journal.pone.0026578

13. **Wirth T, Falush D, Lan RT, Colles F, Mensa P, Wieler LH, *et al***. Sex and virulence in *Escherichia coli*: an evolutionary perspective. *Molecular Microbiology* 2006;60:1136-1151 doi: 10.1111/j.1365-2958.2006.05172.x

14. **Larsen MV, Cosentino S, Rasmussen S, Friis C, Hasman H, Marvig RL, *et al***. Multilocus sequence typing of total-genome-sequenced bacteria. *Journal of Clinical Microbiology* 2012;50:1355-1361 doi: 10.1128/JCM.06094-11

15. **Ingle DJ, Valcanis M, Kuzevski A, Tauschek M, Inouye M, Stinear T, *et al***. *In silico* serotyping of *E. coli* from short read data identifies limited novel O-loci but extensive diversity of O:H serotype combinations within and between pathogenic lineages. *Microbial Genomics* 2016;2:e000064 doi: 10.1099/mgen.0.000064

16. **Gupta SK, Padmanabhan BR, Diene SM, Lopez-Rojas R, Kempf M, Landraud L, *et al***. ARG-ANNOT, a new bioinformatic tool to discover antibiotic resistance genes in bacterial genomes. *Antimicrobial Agents and Chemotherapy* 2014;58:212-220 doi: 10.1128/AAC.01310-13

17. **Carattoli A, Zankari E, Garcia-Fernandez A, Larsen MV, Lund O, Villa L, *et al***. *In silico* detection and typing of plasmids using PlasmidFinder and plasmid multilocus sequence typing. *Antimicrobial Agents and Chemotherapy* 2014;58:3895-3903 doi: 10.1128/AAC.02412-14

18. **Zankari E, Allesoe R, Joensen KG, Cavaco LM, Lund O, Aarestrup FM**. PointFinder: a novel web tool for WGS-based detection of antimicrobial resistance associated with chromosomal point mutations in bacterial pathogens. *Journal of Antimicrobial Chemotherapy* 2017;72:2764-2768 doi: 10.1093/jac/dkx217

19. **Heisig P**. Genetic evidence for a role of *parC* mutations in development of high-level fluoroquinolone resistance in *Escherichia coli*. *Antimicrobial Agents and Chemotherapy* 1996;40:879-885 doi: 10.1128/AAC.40.4.879

20. **Barnard FM, Maxwell A**. Interaction between DNA gyrase and quinolones: effects of alanine mutations at GyrA subunit residues $Ser^{83}$ and $Asp^{87}$. *Antimicrobial Agents and Chemotherapy* 2001;45:1994-2000 doi: 10.1128/AAC.45.7.1994-2000.2001

21. **Wyres KL, Wick RR, Gorrie C, Jenney A, Follador R, Thomson NR, *et al***. Identification of *Klebsiella* capsule synthesis loci from whole genome data. *Microbial Genomics* 2016;2:e000102 doi: 10.1099/mgen.0.000102

22. **Treangen TJ, Ondov BD, Koren S, Phillippy AM**. The Harvest suite for rapid core-genome alignment and visualization of thousands of intraspecific microbial genomes. *Genome Biology* 2014;15:524 doi: 10.1186/s13059-014-0524-x

23. **Bruen TC, Philippe H, Bryant D**. A simple and robust statistical test for detecting the presence of recombination. *Genetics* 2006;172:2665-2681 doi: 10.1534/genetics.105.048975

24. **Stamatakis A**. RAxML version 8: a tool for phylogenetic analysis and post-analysis of large phylogenies. *Bioinformatics* 2014;30:1312-1313 doi: 10.1093/bioinformatics/btu033

25. **Sullivan MJ, Petty NK, Beatson SA**. Easyfig: a genome comparison visualizer. *Bioinformatics* 2011;27:1009-1010 doi: 10.1093/bioinformatics/btr039
