# Supplementary material for: Genomic epidemiology reveals geographical clustering of multidrug-resistant Escherichia coli ST131 associated with bacteraemia in Wales
Source: Nat Commun. 2024 Feb 14;15:1371. doi: 10.1038/s41467-024-45608-1 (PMC10866875; doi:10.1038/s41467-024-45608-1)
Supplement: Supplementary file 3 — Description of Additional Supplementary Files [file 41467_2024_45608_MOESM3_ESM.pdf]

Description of Additional Supplementary Files

File Name: Supplementary Data 1
Description: Whole-genome sequences of 157 Welsh isolates used in this investigation: Metadata and public database accession numbers

File Name: Supplementary Data 2
Description: Quality control metrics for 157 Welsh isolates

File Name: Supplementary Data 3
Description: Quality control metrics for 15 Welsh isolates excluded from this investigation

File Name: Supplementary Data 4
Description: De novo assembly metrics for 142 Welsh isolates

File Name: Supplementary Data 5
Description: Whole-genome sequences of 208 global isolates used in this investigation: Metadata and public database accession numbers

File Name: Supplementary Data 6
Description: Whole-genome sequences of 342 ST131 isolates used in this investigation: Metadata and public database accession numbers

File Name: Supplementary Data 7
Description: Clade C ST131 BEAST analysis results summary
