# Supplementary material for: Genomic epidemiology reveals geographical clustering of multidrug-resistant Escherichia coli ST131 associated with bacteraemia in Wales
Source: Nat Commun. 2024 Feb 14;15:1371. doi: 10.1038/s41467-024-45608-1 (PMC10866875; doi:10.1038/s41467-024-45608-1)
Supplement: Supplementary file 11 — Reporting Summary [file 41467_2024_45608_MOESM11_ESM.pdf]

nature portfolio

Corresponding author(s): Rhys White
Last updated by author(s): Jan 22, 2024

Reporting Summary

Nature Portfolio wishes to improve the reproducibility of the work that we publish. This form provides structure for consistency and transparency in reporting. For further information on Nature Portfolio policies, see our Editorial Policies and the Editorial Policy Checklist.

Statistics

For all statistical analyses, confirm that the following items are present in the figure legend, table legend, main text, or Methods section.

n/a	Confirmed	
☐	☒	The exact sample size (*n*) for each experimental group/condition, given as a discrete number and unit of measurement
☒	☐	A statement on whether measurements were taken from distinct samples or whether the same sample was measured repeatedly
☒	☐	The statistical test(s) used AND whether they are one- or two-sided<br>*Only common tests should be described solely by name; describe more complex techniques in the Methods section.*
☒	☐	A description of all covariates tested
☒	☐	A description of any assumptions or corrections, such as tests of normality and adjustment for multiple comparisons
☐	☒	A full description of the statistical parameters including central tendency (e.g. means) or other basic estimates (e.g. regression coefficient) AND variation (e.g. standard deviation) or associated estimates of uncertainty (e.g. confidence intervals)
☒	☐	For null hypothesis testing, the test statistic (e.g. *F*, *t*, *r*) with confidence intervals, effect sizes, degrees of freedom and *P* value noted<br>*Give P values as exact values whenever suitable.*
☐	☒	For Bayesian analysis, information on the choice of priors and Markov chain Monte Carlo settings
☒	☐	For hierarchical and complex designs, identification of the appropriate level for tests and full reporting of outcomes
☒	☐	Estimates of effect sizes (e.g. Cohen's *d*, Pearson's *r*), indicating how they were calculated

Our web collection on statistics for biologists contains articles on many of the points above.

Software and code

Policy information about availability of computer code

Data collection	Publicly available sequence data was retrieved from the National Center for Biotechnology Information (NCBI) Sequence Read Archive (SRA). This was done using the 'prefetch' and 'fastq-dump' tools within the SRA Toolkit v2.9.0-mac64 (http://ncbi.github.io/sra-tools).
Data analysis	FastQC package v0.11.8 (http://www.bioinformatics.babraham.ac.uk/projects/fastqc/) ; MultiQC v1.7; Kraken v2.0.7-beta; Trimmomatic v0.36; SPANDx v3.2; MGAP (https://github.com/dsarov/MGAP---Microbial-Genome-Assembler-Pipeline); Mauve version snapshot_2015-02-25; QUAST v4.5; MLST v2.19.0 (https://github.com/tseemann/mlst); ABRicate v0.9.7 (https://github.com/tseemann/abricate); PointFinder (https://bitbucket.org/genomicepidemiology/pointfinder/src/master/); Kaptive v0.4; FimTyper 1.0; Parsnp v1.2; RAxML v8.2.10; EasyFig v2.2.2; ART (version ART-MountRainier-2016-06-05); TempEst v1.5.15; BEAST2 v2.6.1; jModelTest v2.1.10; Tracer v1.7.1; FigTree v1.4.4 (http://tree.bio.ed.ac.uk/software/figtree/); EvolView v2

For manuscripts utilizing custom algorithms or software that are central to the research but not yet described in published literature, software must be made available to editors and reviewers. We strongly encourage code deposition in a community repository (e.g. GitHub). See the Nature Portfolio guidelines for submitting code & software for further information.

Data

Policy information about availability of data

All manuscripts must include a data availability statement. This statement should provide the following information, where applicable:

- Accession codes, unique identifiers, or web links for publicly available datasets
- A description of any restrictions on data availability
- For clinical datasets or third party data, please ensure that the statement adheres to our policy

The study sequences are available in the National Center for Biotechnology Information (NCBI) under BioProject accession number PRJNA729115 [https://www.ncbi.nlm.nih.gov/bioproject/?term=PRJNA729115]. Raw Illumina sequence read data have been deposited to the NCBI sequence read archive (SRA (https://www.ncbi.nlm.nih.gov/sra)) under the accession numbers SRR14519411 to SRR14519567 [https://www.ncbi.nlm.nih.gov/bioproject/?term=PRJNA729115]. A complete list of SRA accession numbers is available in Supplementary Data 1 (available in the online version of this article). The high-quality draft assemblies have been deposited to GenBank under the accession numbers JAHBGJ000000000 to JAHBMG000000000, and JAHBRR000000000 to JAHBRT000000000 [https://www.ncbi.nlm.nih.gov/bioproject/?term=PRJNA729115].

Publicly available genome sequence data downloaded for comparative analyses are available in NICU under BioProject accession numbers: PRJDA19053; PRJEA61443; PRJEB2968; PRJNA211153; PRJNA218163; PRJNA307507; PRJNA311313; and PRJNA627752.

The programs used to analyse raw sequence reads for polymorphism discovery and whole-genome sequencing based phylogenetic reconstruction are available as described in the materials and methods. The authors confirm all supporting data and protocols have been provided within the article or through supplementary data files.

Research involving human participants, their data, or biological material

Policy information about studies with human participants or human data. See also policy information about sex, gender (identity/presentation), and sexual orientation and race, ethnicity and racism.

Reporting on sex and gender	Patient sex (male or female) was recorded for each E. coli isolate that was cultured from blood samples.
Reporting on race, ethnicity, or other socially relevant groupings	Reporting on race, ethnicity, or other socially relevant groupings was not relevant to this study.
Population characteristics	Patient age was recorded for each E. coli isolate that was cultured from blood samples.
Recruitment	Patients we not recruited. Microbiology laboratories in Public Health Wales (PHW) and across the Welsh National Health Service (NHS) were asked to submit all E. coli blood isolates from blood samples collected between April 2013 and March 2014, to the national Specialist Antimicrobial Chemotherapy Unit (SACU) at University Hospital Wales
Ethics oversight	This work was undertaken on stored bacterial cultures and no additional clinical samples were collected from any persons to facilitate this study. Patient anonymity was ensured by Public Health Wales' E. coli bacteraemia project manager by preparing a pseudonymised study dataset and samples for whole-genome sequencing and analysis by Cardiff University.

Note that full information on the approval of the study protocol must also be provided in the manuscript.

Field-specific reporting

Please select the one below that is the best fit for your research. If you are not sure, read the appropriate sections before making your selection.

☒ Life sciences ☐ Behavioural & social sciences ☐ Ecological, evolutionary & environmental sciences

For a reference copy of the document with all sections, see nature.com/documents/nr-reporting-summary-flat.pdf

Life sciences study design

All studies must disclose on these points even when the disclosure is negative.

Sample size	Microbiology laboratories in Public Health Wales and across the Welsh National Health Service (NHS) were asked to submit all E. coli blood isolates from blood samples collected between April 2013 and March 2014. Novel AMR profiles and profiles with phylogeography were characterised by polymerase chain reaction (PCR). Selected samples were sequenced based on their determined phylogenetic groups. Sample sizes were determined based on availability on isolates for sequence analysis.
Data exclusions	We identified and excluded the sequence data for 15 Welsh isolates from further analysis based on the sequencing coverage below 20-fold.
Replication	This study involved the collection and genome sequencing of E. coli isolates from blood samples from individuals in Wales between April 2013 and March 2014. Our study was based on a restricted dataset that came about through collaborators at Public Health Wales. Replication is deemed unfeasible due to the time-specific nature of the data collection, making it challenging to recreate identical circumstances. Additionally, the unique sample pool, ethical considerations surrounding human samples, and the complex process of genome sequencing

contribute to the impracticality of reproducing the study under controlled conditions. External factors, such as changes in regulations or research priorities, further hinder the possibility of replication.

Randomization

Randomization was not relevant to this study. The study was observational, focusing on the analysis of naturally occurring events without intervention. Microbiology laboratories in Public Health Wales and across the Welsh National Health Service (NHS) were asked to submit all E. coli blood isolates from blood samples collected between April 2013 and March 2014, to the national Specialist Antimicrobial Chemotherapy Unit at University Hospital Wales.

Blinding

Blinding was not deemed relevant for this genomic surveillance study of E. coli in Wales due to the nature of the research. The focus on objective genomic analysis reduced the possibility for subjective bias. Laboratory processes and data analysis were based on established protocols and computational methods, reducing the need for blinding. This study emphasised on enhanced microbiological surveillance.

Reporting for specific materials, systems and methods

We require information from authors about some types of materials, experimental systems and methods used in many studies. Here, indicate whether each material, system or method listed is relevant to your study. If you are not sure if a list item applies to your research, read the appropriate section before selecting a response.

Materials & experimental systems

n/a	Involved in the study
☒	☐ Antibodies
☒	☐ Eukaryotic cell lines
☒	☐ Palaeontology and archaeology
☒	☐ Animals and other organisms
☒	☐ Clinical data
☒	☐ Dual use research of concern
☒	☐ Plants

Methods

n/a	Involved in the study
☒	☐ ChIP-seq
☒	☐ Flow cytometry
☒	☐ MRI-based neuroimaging

Plants

Seed stocks

Report on the source of all seed stocks or other plant material used. If applicable, state the seed stock centre and catalogue number. If plant specimens were collected from the field, describe the collection location, date and sampling procedures.

Novel plant genotypes

Describe the methods by which all novel plant genotypes were produced. This includes those generated by transgenic approaches, gene editing, chemical/radiation-based mutagenesis and hybridization. For transgenic lines, describe the transformation method, the number of independent lines analyzed and the generation upon which experiments were performed. For gene-edited lines, describe the editor used, the endogenous sequence targeted for editing, the targeting guide RNA sequence (if applicable) and how the editor was applied.

Authentication

Describe any authentication procedures for each seed stock used or novel genotype generated. Describe any experiments used to assess the effect of a mutation and, where applicable, how potential secondary effects (e.g. second site T-DNA insertions, mosiacism, off-target gene editing) were examined.
